# The use of implementation mapping in healthcare settings: a scoping review

**DOI:** 10.3389/fpubh.2025.1603178

**Published:** 2025-07-16

**Authors:** Kym Warhurst, Zephanie Tyack, Michael Beckmann, Bridget Abell

**Affiliations:** ^1^Mater Misericordiae Ltd, Brisbane, QLD, Australia; ^2^Australian Centre for Health Services Innovation and Centre for Healthcare Transformation, School of Public Health and Social Work, Faculty of Health, Queensland University of Technology, Brisbane, QLD, Australia; ^3^Faculty of Medicine, The University of Queensland, Brisbane, QLD, Australia; ^4^Mater Research, The University of Queensland, Brisbane, QLD, Australia

**Keywords:** healthcare, Implementation Mapping, scoping review, implementation science, value-based healthcare

## Abstract

**Background:**

Implementation Mapping is a structured, theory-informed approach designed to facilitate the selection and tailoring of implementation strategies to improve the uptake of healthcare interventions. Despite growing use in healthcare settings since being introduced in 2019, there has been limited synthesis of the application or effectiveness of Implementation Mapping. This scoping review aimed to explore the extent and type of evidence that uses Implementation Mapping methodology to implement programmes or practices in healthcare settings to identify common approaches, benefits, challenges, and future directions.

**Methods:**

A systematic search was undertaken in March 2023 and updated in August 2024 across four electronic databases (MEDLINE, Embase, Scopus and CINAHL) using “Implementation Mapping” as a key word. This was supplemented with citation tracking (including the paper originally describing Implementation Mapping), manual searches of key journals, and a Google scholar search. Studies were included if they reported the use of Implementation Mapping to design and implement healthcare programmes or practices.

**Results:**

The review identified 32 relevant publications, reporting on 29 unique studies, primarily conducted in the United States. Implementation Mapping has been applied across a diverse range of healthcare settings, with common applications in chronic disease management, cancer care, mental health, and allied health interventions. There was notable variation in the application and reporting of Implementation Mapping. The prioritisation of determinants and implementation strategies was inconsistently described, and evaluation of implementation outcomes was often lacking. Despite these challenges, Implementation Mapping was found to provide a structured and participatory approach to implementation planning, promoting stakeholder engagement and the integration of theories, models and frameworks.

**Discussion:**

Implementation Mapping appears to be a valuable tool for guiding the selection and adaptation of implementation strategies in healthcare, but its application remains inconsistent. Greater standardisation in reporting, enhanced methodological guidance, and broader geographic and contextual diversity in studies are needed to maximise its impact. Future research should focus on evaluating the clinical and implementation outcomes associated with Implementation Mapping to establish the effectiveness of this approach in improving healthcare practices. Approaches for prioritising determinants and strategies in Implementation Mapping are suggested based on the findings and other literature.

## 1 Introduction

The translation of new evidence and interventions into clinical practice remains a significant challenge for contemporary, evidence-based healthcare. There is a lag of 15 to 17 years for research evidence to be implemented into clinical practice (1–4). Such delays in knowledge translation may have wide-reaching impacts, both at an individual patient and broader population level, through unrealised potential for improved outcomes, and impacts on health service efficiency and resources. Addressing this knowledge translation gap is essential for the delivery of high-value care.

Implementation science seeks to bridge this gap by improving the application of evidence-based practices. Unlike clinical research, implementation science considers not only the effectiveness of innovations but also the broader clinical context that may impact their uptake. A key feature of this field is using theories, models, and frameworks to understand implementation determinants, processes and outcomes. However, practical guidance about the application of these frameworks to develop and select implementation strategies to improve implementation outcomes (for example, adoption, implementation, sustainment) remains limited (5). Several concerns have been raised in the literature regarding the design and selection of implementation strategies. Issues include limited use of theory-informed planning and strategy selection, failure to clearly define implementation goals, inadequate understanding of the determinants impacting on implementation (which informs strategy selection) and limited understanding and description of the mechanisms of change hypothesised to lead to the desired result (5–8).

Implementation Mapping is an emerging approach informed by the theories, models and frameworks of implementation science and designed to plan and tailor implementation strategies to context. It was first described by a group of American and European authors, with early examples of Implementation Mapping applied in school-based settings in the Netherlands (the “Focus on Strength” programme targeting overweight and obesity in children) (9, 10), and healthcare settings in the United States (the “Peace of Mind” programme to increase mammography screening in low income women) (11). The Implementation Mapping approach evolved from Intervention Mapping, a six-step approach developed for the design and implementation of multi-level health promotion programmes and interventions (12, 13). While Intervention Mapping includes a step focused on planning for adoption and implementation, Implementation Mapping expands on this process by introducing additional structured tasks. These tasks include [1] conducting a needs assessment and identifying adopters and implementers; [2] identifying adoption and implementation outcomes, performance objectives and determinants, and creating matrices of change; [3] choosing theoretical models, selecting or creating implementation strategies; [4] producing implementation protocols and materials; and [5] evaluating implementation outcomes (5). Implementation Mapping was developed as a systematic process to address some of the gaps in the field of implementation science, aiming to optimise implementation of evidence-based interventions through consideration of implementation context, determinants and mechanisms (5). It also provides practical guidance for creating context-specific implementation strategies which enhance implementation efforts and may improve implementation and intervention outcomes (5, 14). Additionally, Implementation Mapping can enhance the understanding of the mechanisms of change for the chosen implementation strategies (5, 14).

Since its introduction in 2019, there has been a rapid uptake of Implementation Mapping in healthcare settings (15–17). However, despite this growing field of literature, there is no systematic or scoping review about this methodology. Moreover, it is not yet clear how Implementation Mapping is being used across different applications and disciplines. A previous systematic review examined the use of Intervention Mapping to enhance health care professional practice but did not include Implementation Mapping (18). That review concluded that Intervention Mapping provides a systematic, theory and evidence-informed framework to guide context specific programme and intervention development and implementation to achieve practice change. However, whether Implementation Mapping offers similar advantages is not yet known.

Synthesising research on Implementation Mapping will provide an understanding of the existing knowledge and methods and the gaps related to the use of this approach. This could include exploring what does and does not work, when it is most useful, frequency of use across different fields or disciplines, and reported fidelity to the methodology. This will inform further research, and support more effective use of Implementation Mapping in healthcare settings. Consequently, this scoping review aims to investigate how Implementation Mapping has been used in programmes and practices in healthcare or hospital settings. Specifically, it seeks to examine the settings, participants, and process involved, and impact on implementation outcomes and patient care.

## 2 Methods

A preliminary search of MEDLINE, the Cochrane Database of Systematic Reviews and JBI Evidence Synthesis was conducted which confirmed no published systematic reviews or scoping reviews on the topic. A scoping review protocol was developed, guided by the JBI methodology (19). A scoping review was chosen as Implementation Mapping is a new process with emerging evidence that is not well characterised. Unlike systematic reviews, scoping reviews allow an exploratory approach for identification and mapping of the existing evidence, highlighting knowledge gaps and important considerations (20, 21). This supports the identification and synthesis of evidence about how Implementation Mapping can be used to inform implementation of programmes or practice changes in healthcare settings rather than evaluating the evidence itself.

Conduct and reporting of the review was guided by the JBI methodology for scoping reviews (19) and the Preferred Reporting Items for Systematic Reviews and Meta-Analyses extension for Scoping Review (PRISMA-ScR) guidelines (21). The review protocol was not registered. Ethical board review was not required as all data was publicly available.

### 2.1 Eligibility criteria

Publications were included in this review if they reported on the use of Implementation Mapping, as described by Fernandez et al. (5), to design implementation strategies to implement programmes or practices in healthcare settings. Study eligibility criteria are outlined in Table 1.

**Table 1:** Inclusion and exclusion criteria.

	Inclusion criteria	Exclusion criteria
Population	Human subjects involved in Implementation Mapping studies in a healthcare context No limitations were applied relating to age, diagnosis or condition of the target population	Nil
Concepts	1. *Implementation Mapping:* Studies reporting the use of “Implementation Mapping.” Studies whose methodology was aligned to the implementation mapping approach as described by Fernandez et al. (5) but did not explicitly use the term “Implementation Mapping.” To be eligible, all studies identified by concepts 1a and 1b must have utilised at least the first four tasks of Implementation Mapping: [1] conducting a needs assessment and identifying adopters and implementers; [2] identifying adoption and implementation outcomes, performance objectives and determinants, and creating matrices of change; [3] choosing theoretical models, selecting or creating implementation strategies; [4] producing implementation protocols and materials. Reporting and completion of Task 2 and Task 3 were considered to be essential elements distinguishing Implementation Mapping from similar approaches when deliberating about eligibility amongst the authors.	Studies which lacked sufficient detail in the paper for the authors to determine whether the reported implementation approach met the Implementation Mapping inclusion criteria and additional information was not available from the authors. Studies which reported Intervention Mapping without Implementation Mapping.
	2. *Healthcare practices OR programs* Healthcare practices were interpreted as specific aspects or activities of care, for example, implementation of a diagnostic test, a surgical procedure or new treatment. Healthcare programs were considered to be a set of related activities, such as a care bundle (of related evidence-informed practices performed collectively) (58), longitudinal programme of care for a condition or implementation of a new model of care. Both patient-facing and provider-facing practices and programs were eligible for inclusion.	
	3. *Intended health benefits to the recipients or the broader population* An additional requirement for inclusion was that implementation of the healthcare practice or programme was intended to provide health benefits to the recipients or the broader population.	
Context	Varied healthcare settings and disciplines were considered, including primary care, community health and allied health practices as well as hospital settings which encompassed secondary and tertiary hospitals, and outpatient and inpatient settings.	All other settings, e.g., education, social services
Limitations	No language limitations were applied. Implementation Mapping was first described in the literature in 2019. Consequently, to capture subsequent studies using this methodology, searches were limited to 2019 onwards.	
Types of sources	Original research studies and systematic or scoping reviews published in peer-reviewed journals were eligible for inclusion. Conference abstracts published in peer reviewed journals were considered for inclusion if there was adequate detail to determine eligibility or additional information could be obtained from the authors to determine eligibility. *Study design:* There were no specifications about the design of original studies (for example randomised controlled trials, cohort, case, observational, cross-sectional, and quasi-experimental studies were eligible) which could include qualitative, quantitative, and mixed-methods approaches.	Books, theoretical articles (i.e., not describing an actual intervention/program), and articles on interventions or programs not related to health care. Protocols describing studies which met the inclusion criteria were excluded unless a full publication reporting the subsequent use of Implementation Mapping proposed in the protocol was identified.

### 2.2 Search strategy and information sources

Preliminary searches of PubMed and Google Scholar were undertaken using the phrase “Implementation Mapping” to identify relevant articles on the topic. The keywords and content of these articles were used to develop a MEDLINE search string in conjunction with a medical librarian, combining keywords and index terms related to “Implementation Mapping.” The final search strategy was reviewed and finalised by KW and BA. The final search strategy included keywords and terms relating to ‘Implementation Mapping’, implementation planning and healthcare. An example of the search strategy for Medline (Ovid) presented in Table 2.

**Table 2:** Final search strategy used to identify articles in Medline (Ovid).

Database	Search string
MEDLINE (Ovid)	“implementation mapping.”af. “strategy mapping.”af. implementation plan*.af. implementation strateg*.af. exp. Hospitals/ exp. Health Facilities/ exp. “Delivery of Health Care”/ healthcare.mp. hospital*.mp. health facilit*.mp. health unit*.mp. ward.mp. clinic.mp. inpatient*.mp. outpatient*.mp. community.mp. health cent*.mp. health system*.mp. 1 or 2 or 3 or 4 5 or 6 or 7 or 8 or 9 or 10 or 11 or 12 or 13 or 14 or 15 or 16 or 17 or 18 19 and 20

Database searches of MEDLINE, Embase, Scopus and CINAHL were performed using this search string, with adaptation for each database and/or information source. Searches were conducted in March 2023 and updated in August 2024 (date last searched 5th August 2024). A manual search was conducted within specialist implementation science journals (Implementation Science and Implementation Science Communications) and Frontiers in Public Health using the term “Implementation Mapping.” The reference lists of the included studies were reviewed to identify additional relevant citations. Forward citation checking of the final included references from both the database and manual journal searches was also conducted using the Systematic Review Accelerator (22), with de-duplication and screening of additional references using the same approach as the references from the original database searches. This was supplemented with a manual search of the first 10 pages of Google Scholar in October 2024 using the search term “Implementation Mapping” to identify any alternative sources of potentially relevant unpublished studies, conference abstracts and grey literature (such as reports by government or non-governmental organisations, policy documents, conference proceedings, theses and dissertations).

#### 2.2.1 Study/source of evidence selection

Following the searches, all identified citations were collated and uploaded into The Systematic Review Accelerator (SRA) (22) and duplicates were removed using The Deduplicator (23). Titles and abstracts were then screened against the inclusion criteria. KW and BA independently reviewed the same 200 publications (2.5% of de-duplicated records), reconciled any differences and finalised the inclusion and exclusion criteria. KW reviewed the title and abstract of remaining publications followed by full text review and assessment of potentially relevant publications against the pre-defined inclusion criteria. At all stages of the screening and selection process KW and BA discussed any sources of evidence which could not be easily categorised and agreed on the final decision to include or exclude. Reasons for exclusion of sources of evidence at full text review stage were recorded and reported in the scoping review.

The results of the search and the study inclusion process are reported in full and presented in the PRISMA-ScR flow diagram (21).

#### 2.2.2 Data extraction

Included publications were analysed using Microsoft Excel and Word. A data extraction form was developed and tested by the authors after finalisation of the search strategy and search terms. Minor iterative revisions were made to the form throughout data extraction as deemed necessary by all authors. Final data points are provided in Table 3. Data extraction was performed by KW. The reviewers discussed and resolved any questions or irregularities throughout data extraction.

**Table 3:** Data extraction fields.

Publication details	Project details	Implementation Mapping approach
Authors Publication year Journal (or conference) Citation details	Country/state/city where the project was conducted Objectives Participants (target population) Participants (organisational), Implementation stakeholders Concept (the practice or programme to be implemented) The nature of the intervention (clinical or public health) Context Setting	Approach to Implementation Mapping (for each task) as described by the authors: Was it done How was it done What theories, models or frameworks were used Findings and/or outcome Findings/outcomes Challenges/limitations Implications

Authors of included or potentially included publications were contacted to request missing or additional data where required (including to determine eligibility), particularly for abstracts or posters and for projects which were noted to be ongoing.

#### 2.2.3 Data analysis and presentation

We analysed the studies and report the results in alignment with the review objective and the review questions: firstly, how has Implementation Mapping been used to implement programmes or practices in healthcare settings (settings, participants, process); and secondly, what is the impact of Implementation Mapping on implementation outcomes and patient care? Qualitative content analysis was used for analysis and interpretation of qualitative data.

Data is presented graphically, diagrammatic or in tabular form. Additionally, a narrative summary describes how the results relate to the reviews objective and question/s. Qualitative and quantitative results are presented separately.

## 3 Results

### 3.1 Selection of sources of evidence

**Figure 1:**
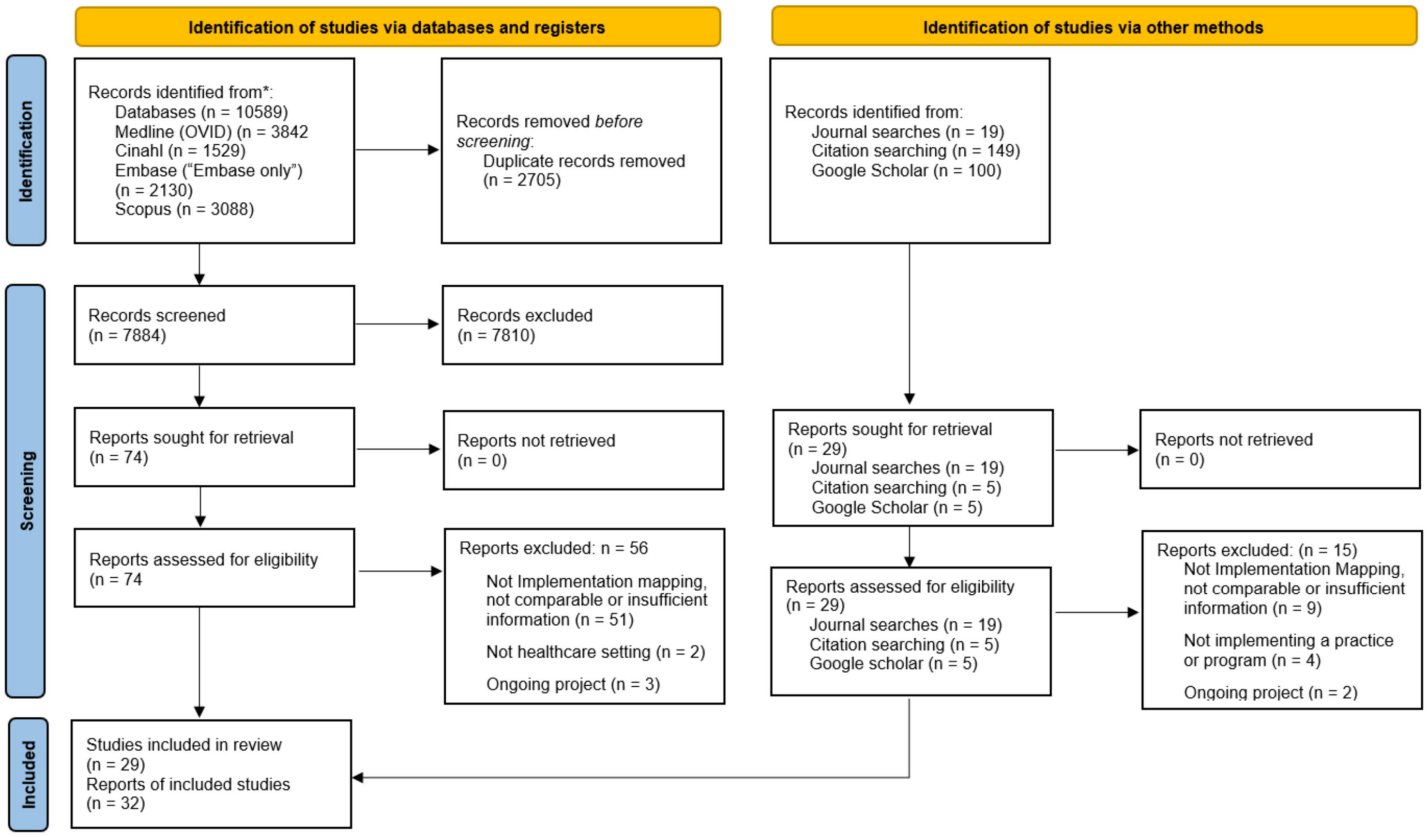
PRISMA flow diagram.

Thirty-two relevant publications were identified, reporting on 29 different studies/projects (Figure 1). Database searches revealed a total of 7,884 publications, of which 74 were potentially relevant and retrieved for full text review, resulting in identification of 18 publications and 15 projects which met inclusion criteria. Three projects had two publications for the same project. An additional 28 potentially relevant records were identified through other methods (journal searches, citation searching and Google Scholar), with inclusion of a further 14 studies. Seventy-one records were excluded following full-text review (56 from original searches and 15 from studies identified by other methods). The majority of these (60 of 70) did not report on the use of Implementation Mapping, were not sufficiently comparable to the Implementation Mapping process (as described by Fernandez et al.) or did not have sufficient information about the Implementation Mapping process to meet the stated eligibility criteria.

Additional information was obtained from the authors for two of the 29 projects included in this review (24, 25). All other data was obtained from the published peer-reviewed articles included in the review.

### 3.2 Characteristics of sources of evidence

The Implementation Mapping approach was applied to a wide variety of concepts and healthcare interventions with a diverse range of target healthcare practitioners and patient populations. The characteristics of the included studies are outlined in Supplementary Table 1.

The majority of projects were conducted in The United States of America (25 of the 29 projects), with Australia, The United Kingdom, Mozambique and The Netherlands each reporting one Implementation Mapping project.

Eighteen projects implemented a clinical intervention, addressing issues such as medication adherence for oral anticancer agents (26), and person-centred goal setting and goal management in rehabilitation (27, 28). Public health-focused interventions were reported in 11 projects, with examples including prevention of diabetes (29) and HIV (25).

The settings for the included studies were diverse, and some were difficult to clearly categorise. Thirteen (out of 29) were undertaken in a primary or community care setting, six in specialist outpatient clinics, two in multiple settings (hospital and clinic), one in long term residential care, one in community pharmacy and one in rehabilitation. While authors of eight projects reported their projects were conducted in a hospital-based setting, this was not clearly reported in many other projects. Furthermore, application across inpatient or outpatient hospital settings was not consistently described.

The Implementation Mapping approach was applied in a diverse array of healthcare areas. Cancer care was the most reported field in which Implementation Mapping was used (6 projects), followed by chronic pain (4 projects), mental health (3 projects) and pharmacy/medication safety (3 projects). Two projects were reported in the areas of HIV care, geriatrics, rehabilitation, critical care (intensive care and emergency medicine), and chronic medical conditions (diabetes and hypertension). A single Implementation Mapping project was reported in paediatrics, substance use (tobacco), and maternity care. Projects in the area of allied health featured commonly, reported in almost half of the included projects (14 of 29 projects) (15–17, 26–38), with physical therapy interventions (including physiotherapy and occupational therapy) reported in four projects (27, 28, 35, 36, 38).

### 3.3 Results of individual sources of evidence

**Figure 2:**
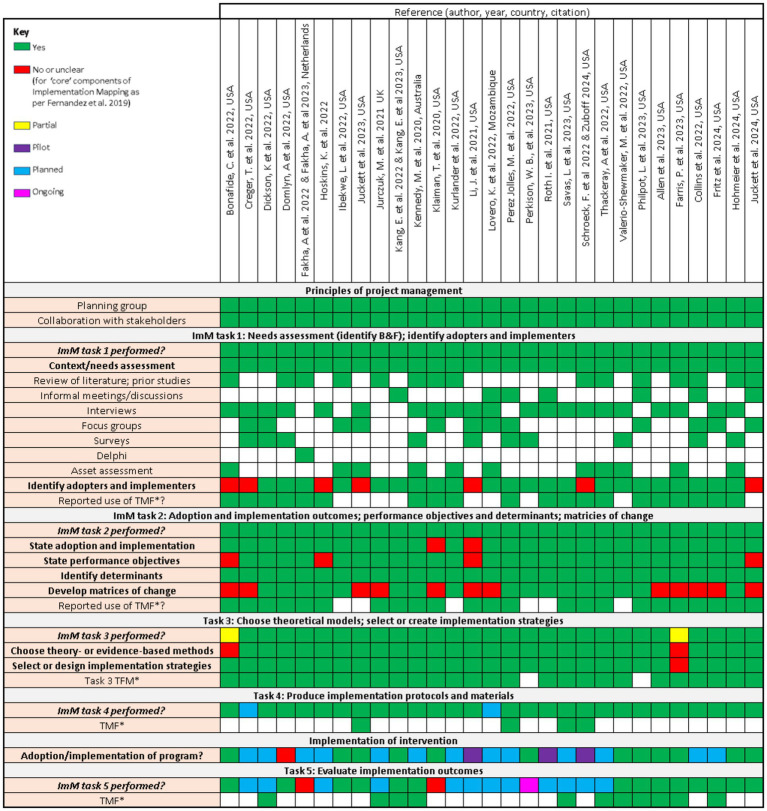
Approaches to Implementation Mapping, use of theories, models, frameworks and tools in included studies. B, barrier; F, facilitator; TMF, theory, model or framework. ^*^Theories, models and frameworks may also include methods and tools.

The approach to the Implementation Mapping tasks, key points and use of theories, models and frameworks (TMF) for the included studies are outlined in Figure 2.

#### 3.3.1 Task 1: needs assessment (identify barriers and facilitators); identify adopters and implementers

Literature reviews or studies conducted prior to Task 1 (preceding studies) were reported in 15 projects (52%). All projects described the use of interviews, focus groups and/or surveys as part of the context assessment. Eleven projects (38%) reported assessment of assets (resources, existing processes, baseline data). Identification of adopters and implementers was clearly stated in 22 of 29 projects and was implied in most reports. Questions were used to assist the identification of adopters and implementers, such as *“who will implement”* (15, 27, 28), and *“will different people need to implement different components?”*(15).

Several projects reported the use of rapid qualitative analysis (25, 34) or rapid analytical methods/techniques (39–41), content analysis of qualitative data (32), or thematic analysis of interview data using the Consolidated Framework for Implementation Research (CFIR) (17, 30, 31, 35, 37, 39, 42–44).

*Task 1 TMFs and tools*

The Consolidated Framework for Implementation Research (CFIR) was the most reported TMF utilised in Task 1 (15 of 29 studies) (17, 24–26, 30, 31, 35–37, 39–45). Other reported TMFs were Reach, Effectiveness, Adoption, Implementation, Maintenance (RE-AIM) (16), the Theoretical Domains Framework (TDF) (33), Health Equity Implementation Framework (HEIF) (39), Exploration Preparation Implementation Sustainment (EPIS) (46), Social Cognitive Theory (SCT) (34), Interactive Systems Framework (ISF) (34), R-MC2 (readiness = motivation × innovation specific capacity × general capacity) (34), and Tailored Implementation for Chronic Diseases Framework (TICD) (47, 48), each of which were reported once. Two projects describe the use of multiple TMFs for Task 1 (34, 39). The TMFs used in Task 1 are outlined in Table 4.

**Table 4:** Prioritisation of implementation strategies.

Approach to prioritisation	How this was done	References
Collaborative approaches	Stakeholder engagement, consultation with experts, meetings, focus groups, workshops, feedback, co-creation, surveys, interviews, nominal group techniques Iterative refinement for final selection of implementation strategies	All included studies
Evidence-informed selection	Behaviour Change Taxonomy ERIC Compilation Behaviour Change Wheel CFIR-ERIC strategy tool Waltz’s Barrier-to-strategy matching tool Evaluation of literature for evidence of relative effectiveness	(15, 16, 27, 28, 32, 35, 38, 42, 49, 52)
Informed by CFIR and context	Focused on methods and strategies that would address determinants across multiple inner context levels, considered complementary with existing strategies	(39, 46)
	One study reported this was due to the nature of the innovation and intervention context	(46)
Decision matrix	*Effort versus impact*One project extended this to consider: Broad vs. narrow scope (based on number of change objectives addressed) Quantitative assessment of required time commitment from local staff Likely impact of strategy in a clinical setting	(40, 47, 48)(47, 48)
	*Relevance and feasibility*	(34)
	*Ranking: importance vs feasibility*Proposed implementation strategies were ranked by being: Important and feasible Important but not feasible Feasible but not important Not important, not feasible	(50)
Combined approach to prioritisation	Final selection of core strategies based on four elements: Empirical evidence of effectiveness Support by relevant theory of change Pragmatic rationale (feasibility, importance, practicality, applicability to context) Feedback following consultation	(30, 31)

#### 3.3.2 Task 2: adoption and implementation outcomes; performance objectives and determinants; matrixes of change, i.e., state steps to implementation (who needs to do what to ensure the intervention is adopted, implemented and maintained?)

Task 2 was performed in all studies, and identification of determinants was universally described in all projects. While most studies reported adoption and implementation outcomes, performance objectives, and development of matrices of change, in some reports it was unclear whether this had been done and if so, how this had been achieved.

The use of questions to guide the steps for Task 2 was reported for eight projects (15, 17, 26–29, 34, 37, 43). Questions such as *“who needs to do what to ensure the program is adopted”*(29) were used for adoption and implementation outcomes, while *“what do the cancer specialists and navigators need to do to accomplish each of these outcomes?”* (15) guided performance objectives.

The development of matrices of change was most conducted by crossing performance objectives (rows) with determinants (columns) to create a matrix of change objectives. In some projects these were stratified by roles/actors or implementation science theories, models or frameworks (for example CFIR, Social Cognitive Theory, Theoretical Domains Framework, TICD framework, EPIS phases). An additional question, *“what has to change in the determinant/s in order to accomplish this implementation performance objective”* was used to aid development of the matrix of change objectives. The resulting change objectives subsequently informed selection or development of implementation strategies in Task 3.

*Task 2 TMFs and tools*

Over three quarters of projects (23/29) utilised a TMF for Task 2. CFIR was most commonly used (13 projects) (25–28, 36–41, 44, 49, 50), followed by behaviour change taxonomy (BCT) (25, 27, 30, 31), Proctor’s implementation research framework (27, 28, 38, 50), and SCT (27, 28, 34, 36) with each used in 3 projects. Expert Recommendations for Implementing Change (ERIC) (25, 51), logic model (24, 35) and EPIS (34, 46) were each used in 2 projects. Only single studies reported use of the TDF (16), ISF (34), TICD (47, 48), RE-AIM (45), Implementation Research Logic Model (IRLM) (41), and Health Equity Implementation Framework (HEIF) (39). Nine projects reported the use of more than one TMF for Task 2.

##### 3.3.2.1 Prioritisation of determinants

Prioritisation of determinants was reported in six projects (21%) (15, 17, 29, 35, 44, 52). Four projects described consideration of importance and changeability when prioritising determinants (15, 26, 29, 44), one prioritised determinants which were considered critical to successful implementation (17), and one project used an evidence-informed approach with findings of a literature review of the implementation of similar programmes informing the prioritisation of determinants (52). The study by Domlyn et al. identified prioritisation of change objectives as an essential element, proposing this should be added to the Implementation Mapping process (40).

#### 3.3.3 Task 3: choose theoretical models; select or create implementation strategies. Relevant behavioural theories are identified. Implementation team select or design implementation strategies to build on the selected theories to overcome barriers and identify the steps to implementation

While Task 3 was performed in all reported projects, the description of the various steps for Task 3 (state adoption and implementation outcomes, state performance objectives, identify determinants, develop matrices of change) and how these steps were operationalised was often only implied or unclear.

Most projects reported development of a proposed list of theoretical change methods and implementation strategies informed by the findings of Tasks 1 and 2. Generally this was done collaboratively between the researchers, planning group, advisory group and/or stakeholders.

*Task 3 TMFs and tools*

The use of theories, models and frameworks was nearly universally reported for Task 3 (27 of 29 projects), with 16 studies reporting the use of more than one implementation science-informed theory, model or framework. Many TMFs were utilised for Implementation Mapping Task 3, with eight approaches reported more than once: ERIC (11 projects); CFIR (6 projects); CFIR-ERIC matching tool and SCT (5 projects each); Behaviour Change Wheel, Logic Model, Taxonomy of Behaviour Change Methods (3 projects each); and Diffusion of Innovations theory (2 projects). A further 18 approaches were reported once each and are listed in Table 5.

**Table 5:** Theories, models, frameworks and tools used to support Implementation Mapping in all included studies.

	Number of projects reporting	Task 1	Task 2	Task 3	Task 4	Task 5
Theories, models and frameworks						
Consolidated Framework for Implementation Research (CFIR)	20	15	13	11		1
Proctor’s implementation research framework	6		3	1		3
Social Cognitive Theory (SCT)	5	1	3	4	1	1
RE-AIM	5	1	1			5
Behaviour Change Wheel	3			3		
Weiner et al. (59) (Feasibility, acceptability, appropriateness)	3					3
Exploration, Preparation, Implementation, Sustainment (EPIS)	2	1	2	1	2	
Theoretical Domains Framework (TDF)	2	1	1			
Diffusion of innovations	2			2		
Health Equity Implementation Framework (HEIF)	1	1	1	1		1
COM-B	1			1		
Interactive Systems Framework (ISF)	1	1	1			
Tailored Implementation of Chronic Diseases (TICD)	1	1	1			
PARIHS framework (Promoting Action on Research Implementation in Health Services)	1			1		
Theory of change	1			1		
Organisational development^*^	1			1		
Organisational level^*^	1			1		
Social network^*^	1			1		
Adult learning^*^	1			1		
Social learning^*^	1			1		
Systems science approach^*^	1			1		
Tools, guides or processes						
Expert Recommendations for Implementation of Change (ERIC)	15		2	15		
CFIR-ERIC matching tool	6			6		
Logic model	3		2	3		1
Proctor’ recommendations for specifying and reporting implementation strategies	3				2	1
Implementation Research Logic Model (IRLM)	2		1	1		1
Nominal group technique	1			1		
Strengths, Weaknesses, Opportunities, Threats (SWOT) analysis^*^	1		1	1		
Political, Economic, Social, Technological, Legal, Environmental (PESTLE) analysis^*^	1		1	1		
Taxonomy, lists etc						
Behaviour Change Taxonomy (BCT)	3		3	1		
Kok Taxonomy of behaviour change methods	2			2		
EPOC taxonomy (Effective Practice and Organisation of Care)	1			1		

*Denotes approaches adopted from fields or disciplines beyond Implementation Science.

##### 3.3.3.1 Task 3 prioritisation of strategies

Guidance for the selection of implementation strategies to address determinants of implementation is a known gap in the implementation science literature. Prioritisation of implementation strategies was described in 19 projects (15, 16, 26–28, 30–35, 38–42, 46–50, 52).

Varied approaches to the selection and prioritisation of implementation strategies were reported in the included studies, which can be grouped into five methods (Table 4). While not a formally described step for Task 3 of Implementation Mapping, prioritisation of implementation strategies was performed in approximately two thirds of projects.

#### 3.3.4 Task 4: produce implementation protocols and materials

Task 4 was performed in 27 projects, with the remaining two projects reporting that this was planned. Many reports lacked detail of how Task 4 was performed. Most described (or implied) a collaborative approach to production of implementation protocols and materials. Resources were developed by a small group or single individual in some projects, while others reported division of tasks among working groups and allocated specific tasks to relevant experts (for example development of digital resources). Materials were then shared more broadly with planning groups and/or stakeholders for consultation, testing and endorsement.

*Task 4 TMFs and tools*

The use of TMFs was infrequently reported for Task 4, occurring in only 4 of the 29 projects. Two projects used Proctor’s recommendations for specifying and reporting implementation strategies to operationalise implementation strategies, considering the seven dimensions: actors, actions, action targets, outcome, rationale (justification), temporality, dosage (32, 47, 48). Two projects used the EPIS framework (34, 46), and Social Cognitive Theory was reported in one project (34).

#### 3.3.5 Implementation of programmes

Implementation of the intervention was reported for 15 projects, with 3 of these describing a piloting process. A further 13 reported that implementation was planned. One project (40) was not implemented due to contextual changes during the Implementation Mapping process. This was mostly attributed to impacts of the COVID-19 pandemic, with project timeframe and funding limitations contributing to a reduction in executive leadership support for the intervention despite initial optimism regarding feasibility and acceptability. The authors highlighted the significant impact that changes in the outer context can have on the inner context for project implementation and suggested that reassessment of determinants and change objectives at regular intervals during the process of Implementation Mapping may be beneficial.

#### 3.3.6 Task 5: evaluate implementation outcomes

Completion of Task 5, evaluate implementation outcomes, was variably reported for the included projects. Evaluation was performed for 14 projects, planned in a further 12 projects, not stated in 2 and ongoing for one project.

Most projects reported (or planned to evaluate) a combination of clinical outcomes and implementation outcomes. Various approaches were described or planned. Mixed methods evaluation of qualitative and quantitative outcome data was a commonly described approach (15, 16, 25–29, 39, 45, 46, 50, 51). Pilot implementation trials (24, 33) and hybrid trials (41, 46) were also described. For some projects, development of implementation evaluation plans occurred concurrently while developing the intervention (25).

Qualitative outcome data was commonly obtained (or was planned to be obtained) through interviews and/or survey responses from stakeholders, clinicians, relevant healthcare staff and/or consumers. Quantitative data sources were diverse and generally specific to the intervention and context for the individual projects. Reported sources of quantitative data included clinical records (including electronic health records), audits, reports, evaluation of resource use and clinical utilisation.

*Task 5 TMFs and tools*

Eleven projects reported the use of theories, models, frameworks or tools. RE-AIM was reported in five projects (16, 34, 44, 45, 52), Proctor’s implementation research framework in three projects (27, 28, 36, 44), Weiner’s measures of feasibility, acceptability and appropriateness in three projects (26, 35, 39), with one project each reporting the use of Social Cognitive Theory (34), Logic Model (34) and Implementation Research Logic Model (44). The use or combination of more than one TMF for Task 5 was reported in two projects (34, 44).

#### 3.3.7 Use of theories, models and frameworks throughout implementation mapping

Many implementation science theories, models and frameworks were reported in the included projects. Commonly used TMFs include the CFIR, Proctor’s implementation research framework, SCT and RE-AIM. Many TMFs were only used once, particularly for Task 3. Implementation science tools, processes and taxonomies were also commonly used, with common examples including ERIC, CFIR-ERIC strategy matching tool, Proctor’s recommendations for specifying and reporting implementation strategies and implementation research logic model. Some approaches were noted to have been adopted from fields or disciplines beyond Implementation Science. Table 5 provides an overview of the use of TMFs, tools, taxonomies and processes, and approaches adapted from other disciplines in the included studies.

### 3.4 Synthesis of results

#### 3.4.1 How has implementation mapping been used?

The Implementation Mapping approach, first reported in 2019, has been applied to a wide range of interventions in varied healthcare settings. To date, use of this approach has been mostly reported in the United States of America, with almost two thirds of reported projects implementing a “clinical” intervention and approximately one third having a “public health” focused intervention.

The application of the Implementation Mapping approach was highly varied, generally in response to the intervention and implementation context. The specific method used for the various Implementation Mapping tasks was often not clearly described. Stakeholder engagement and collaborative approaches were universally reported.

**Figure 3:**
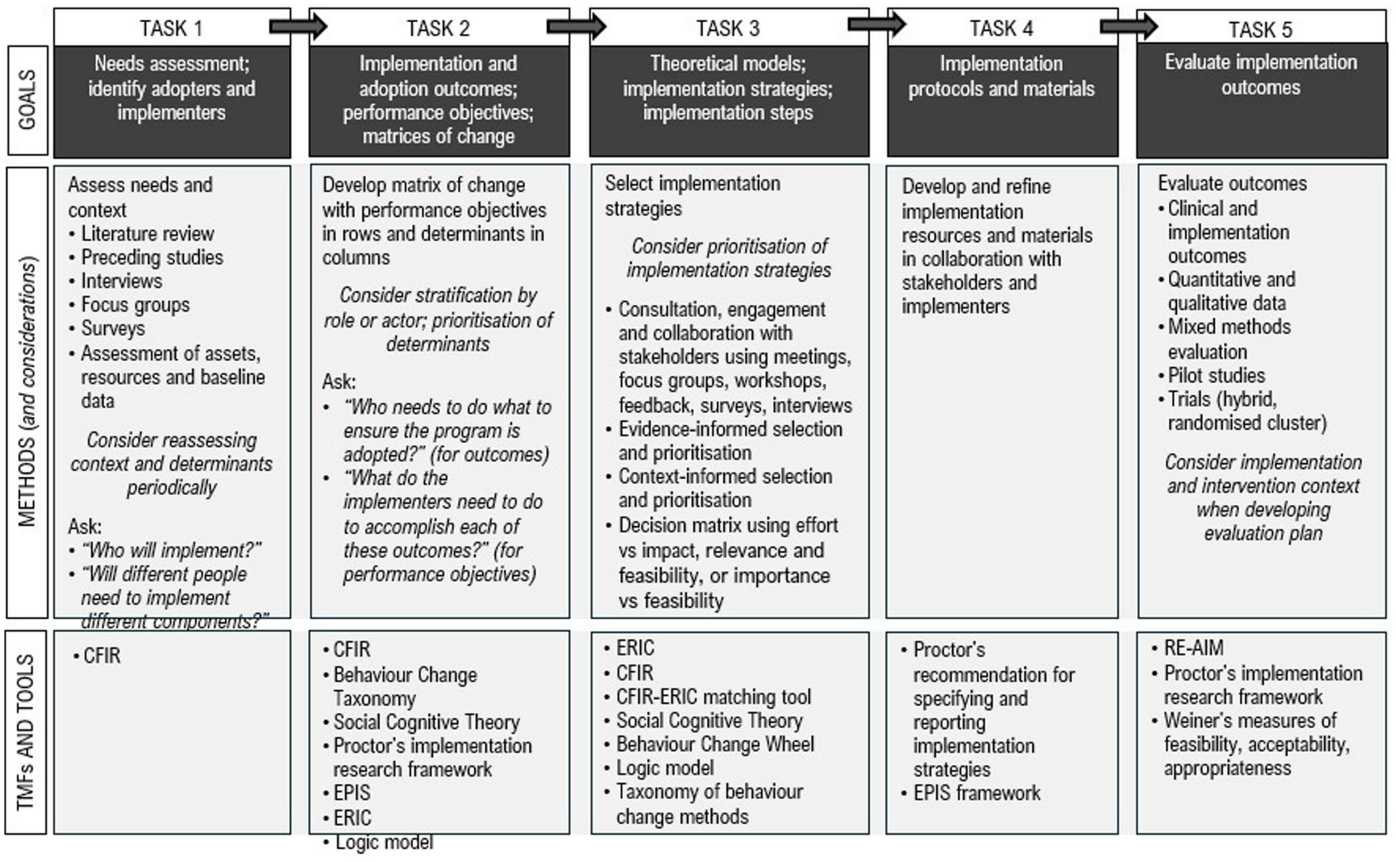
Synthesis of evidence-informed considerations when applying Implementation Mapping in healthcare settings. CFIR, Consolidated Framework for Implementation Research; EPIS, Exploration Preparation Implementation Sustainment; ERIC, Expert Recommendations for Implementing Change; RE-AIM, Reach, Effectiveness, Adoption, Implementation, and Maintenance; TMF’s, theories, models, and frameworks; 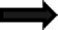 informed by the preceding tasks.

An overview of practical considerations when applying the five tasks of Implementation Mapping is presented in Figure 3. These considerations are summarised from the results and discussion sections across the papers for the included studies.

#### 3.4.2 What is the impact of implementation mapping on implementation outcomes and patient care?

Commonly reported implementation outcomes included reach, adoption, maintenance, acceptability and fidelity. Implementation and maintenance were often reported at baseline (prior to implementation) and several time-points after implementation. Evaluation of effectiveness, when reported, mostly considered process outcomes rather than clinical outcomes. The impact of the Implementation Mapping approach on implementation outcomes was not formally evaluated in any of the included studies. However, multiple authors suggested that the use of Implementation Mapping helped to improve the selection and tailoring of context-specific implementation strategies and implementation planning, thereby improving the effectiveness of implementation. None of the papers provided a solid assessment of how Implementation Mapping impacted on clinical outcomes and patient care.

## 4 Discussion

This review collates the existing literature about the use of Implementation Mapping to implement practices or programmes in healthcare settings. Implementation Mapping has been applied across a diverse range of healthcare settings and intervention types, though with notable variability in its application and reporting. It has primarily been implemented in the United States, with applications in other geographic regions limited, suggesting a concentration of expertise and uptake in North American healthcare settings. Stakeholder engagement and collaborative approaches were universally reported, reinforcing the participatory nature of Implementation Mapping. Additionally, most studies incorporated theories, models, and frameworks, particularly CFIR and ERIC, either overall or to guide determinant analysis and strategy selection, demonstrating appropriate application of Implementation Mapping as a theory-informed approach. However, inconsistent reporting of specific tasks, particularly those in later-stages such as evaluation (Task 5), highlights an area requiring further improvement. Additionally, while prioritisation of determinants (Task 2) and implementation strategies (Task 3) was inconsistently reported, several authors emphasised the importance of prioritisation in guiding effective implementation efforts.

Implementation Mapping was first formally described in 2019 in an attempt to progress the field of implementation science and close the evidence-practice gap by using a systematic process for planning and selecting implementation strategies. The need for systematic approaches to implementation strategy selection has previously been emphasised (7). Our findings suggest that Implementation Mapping meets this need by providing a structured framework applicable across different contexts and implementation stages. Several elements were described across many of the included projects and could therefore be considered the core components of Implementation Mapping. These included the importance of *understanding the implementation context, use of questions* to help frame specific Implementation Mapping Tasks, the use of *implementation science theories, models or frameworks,* and *prioritisation of determinants* (in Task 2) *and implementation strategies* (Task 3). Using these components routinely may improve the Implementation Mapping process, however it is yet to be determined whether these components impact the effectiveness of Implementation Mapping.

Despite its supportive structure, practical application of Implementation Mapping varied widely. The included projects demonstrated many different approaches to Implementation Mapping Tasks, yet most reported that adoption of the Implementation Mapping methodology helped to improve the implementation processes for their projects. Consequently, it is likely that there is no ‘right way’ or ‘best way’ to approach Implementation Mapping. A flexible approach, informed by the nature of the intervention and the implementation context, is likely best when considering application of the Implementation Mapping process, although as discussed there may be core components that should be considered. This approach also aligns with one of the important underlying concepts of Implementation Mapping, the importance of understanding and tailoring to the implementation context (5). Many authors reported the development of more nuanced implementation strategies (responding to key determinants), and implementation plans because of detailed contextual assessment. Furthermore, Domlyn et al. (40) emphasised the role of re-evaluation of the implementation context, particularly when the outer context is changing unexpectedly or rapidly. Knowledge of this can inform healthcare planners and implementers considering this approach and may help optimise future Implementation Mapping activities.

*Prioritisation of both determinants* (in Task 2) *and implementation strategies* (in Task 3) was commonly reported, although this step was not included in the original description of Implementation Mapping (5) nor subsequent editorial by Fernandez and colleagues (14). Domlyn et al. (40) argued that prioritisation of determinants (Task 2) was essential and proposed that this step should be added to the Implementation Mapping process. Almost two-thirds of the included projects reported a process for prioritising implementation strategies, with five different methods of prioritisation described. This suggests that prioritisation of implementation strategies is an important consideration for many projects and may be particularly important for projects undertaken on a larger scale, or with more complex implementation. The ‘combined’ approach described by Fakha et al. (30, 31) which considers empirical evidence of effectiveness, supported by a relevant theory of change (explaining how and why a change is expected to occur), a pragmatic rationale (feasibility, importance, practicality, applicability to context) and feedback following consultation is likely the most comprehensive approach to prioritisation of implementation strategies, yet may not be feasible or necessary in all implementation contexts.

Prioritisation is not a new concept in the field of implementation science, although evidence-based guidance about where or when this is beneficial and how to approach this is limited. In their 2017 systematic review of Intervention Mapping to enhance healthcare professional practice, Durks and colleagues suggested that determinants should be prioritised based on their relevance and changeability (18). A scoping review of prioritisation processes for programme implementation and evaluation in public health highlighted the complexity of prioritisation (53). They found that formal frameworks were seldom utilised and have rarely been used beyond a single study. The authors suggested the development of a prioritisation framework to address this evidence gap.

Prioritisation approaches from related disciplines such as quality improvement and behaviour change science may guide how this could be achieved. A 2001 report describes ranking of improvement initiatives and recommendations according to pre-specified criteria such as (i) the potential impact, (ii) the strength of evidence, (iii) issues relating to implementation; or (a) importance, (b) scientific soundness, (c) feasibility (54). A related approach described more recently in the behavioural change literature uses a matrix to visually appraise impact of the proposed technique or strategy and likelihood of adoption (effort required), with both measures ‘scored’ as high or low (55). The resulting matrix provides an easily understood summary of potential strategies or techniques, which can be considered as *‘easy and effective’* (the ‘low hanging fruit’, generally first priority), *‘hard and ineffective’* (generally low priority) and two intermediate categories, *‘easy but ineffective’* (while low impact, these can have a ‘foot-in-the-door effect which may improve uptake of or challenging techniques or strategies) and *‘hard but effective’* (potential logistical or resource requirements) (55). Methods like these prioritisation approaches were observed in many of the Implementation Mapping studies in this review, however the additional element of context was often considered. Consequently, we suggest the most important considerations for prioritisation of determinants and strategies in Implementation Mapping are selecting a prioritisation approach that is contextually appropriate, pre-specifying the criteria or parameters that will be used, and clearly reporting prioritisation processes and outcomes.

### 4.1 Proposed benefits of implementation mapping

Firm conclusions about the impact of the Implementation Mapping approach on implementation and clinical outcomes cannot be drawn from the included studies. This is consistent with broader concerns in implementation science about the link between use of TMFs and measurable outcomes (56). However, many observed benefits of the methodology were described. The identification and understanding of determinants allowing for data-driven and theory-informed selection of implementation strategies to systematically address major barriers (often across multiple levels) and enact meaningful change was a prominent theme. Implementation mapping provided an over-arching structure or ‘roadmap’ for implementation and allowed for easy incorporation of additional theories, models and frameworks. The critical importance of developing relationships and engaging with stakeholders, clinicians and consumers was highlighted. Overwhelmingly, authors concluded that Implementation Mapping had a beneficial impact on their projects, improving the implementation of practices or programmes in healthcare settings. However, assessing not only implementation success but also clinical and patient-reported outcomes will help determine Implementation Mapping’s broader effectiveness.

### 4.2 Reported limitations of implementation mapping

Several challenges and limitations were reported in the included studies which may hinder the broader adoption of Implementation Mapping. Some were related to the methodology itself and others arose due to the interventions, or the implementation context involved. Implementation Mapping is a resource-intensive process, requiring significant time, expertise, and stakeholder engagement, which may not be feasible in all settings, particularly in low-resource contexts (25, 39, 47, 48). Additionally, translation of implementation science terminology and taxonomies to stakeholders who are unfamiliar with these approaches was also an identified challenge (15, 41). However, as seen in some studies, the use of questions to help frame specific Implementation Mapping tasks may help to focus activities and outputs where implementers or stakeholders are unfamiliar with implementation science approaches and terminology. Simplified language, structured questions to focus tasks, and training in the process could enhance use. Finally, changes in the outer implementation context were commonly reported, most notably related to the unanticipated and profound impact of the global COVID-19 pandemic. However, while these may have impacted intervention implementation, outer context changes were not a limitation of the Implementation Mapping process itself. Regular re-evaluation of determinants and strategies may help mitigate these challenges (40).

### 4.3 Strengths and limitations of the review

To our knowledge, this is the first scoping review to explore the use of Implementation Mapping to implement practices or programmes in healthcare settings. There are several notable limitations to this review. While our search methodology was extensive, there may be other relevant studies which were not identified. Data collection was performed by a single reviewer (KW) with queries resolved in collaboration with BA, ensuring consistency of data extraction. The use of clear definitions of inclusion and exclusion criteria, and a pre-specified data collection template prior to commencement of data extraction likely minimised discrepancies in data collection.

The included studies reported implementation of a wide range of interventions in diverse settings and contexts, and outcome data should be interpreted with caution. As most of these studies were conducted in the US it is difficult to draw firm conclusions about broader applicability. The included studies report the use of Implementation Mapping in a wide variety of settings, although low-resource settings are difficult to clearly identify. It is likely that the elements of Implementation Mapping are translatable to low-resource settings and future efforts to understand this (or clearly report this if it is already occurring) may be beneficial.

Reporting of approaches to the Implementation Mapping tasks was often vague. Specific tasks and sub-tasks were often implied or not clearly described, leading to difficulty in fully understanding the approaches used. We cannot draw conclusions about the importance of following the described sequence of tasks and processes described for Implementation Mapping, nor whether omitting some steps adversely impacts the effectiveness of the Implementation Mapping approach, as these were not evaluated in any of the studies.

The CFIR was the most common TMF used in the included studies. The CFIR is a comprehensive, widely used framework to understand complex contextual factors impacting on implementation and sustainability of implementation, and therefore aligns particularly well with the first two Implementation Mapping Tasks. The ERIC and CFIR-ERIC strategy matching tool were also frequently used, most notably for Task 3, to develop and design implementation strategies that are appropriately tailored to the implementation context, likely reflecting the common use of CFIR in implementation research more broadly (57). This synthesis is not intended to be prescriptive regarding the use of TMF for Implementation Mapping in healthcare settings, but rather reflects the TMF used in the included papers. Tailoring to context may benefit from the use of other theories, models and frameworks not reported in papers included in this review.

Finally, the included studies did not provide sufficient evidence to draw firm conclusions about the impact of Implementation Mapping on clinical and intervention outcomes.

### 4.4 Future considerations

Future research should explore how Implementation Mapping can be adapted for different healthcare systems and settings, particularly in low-and middle-income countries. More comparative studies are needed to determine optimal TMF combinations for different contexts. Simplified or resource-adapted versions of Implementation Mapping may improve feasibility in time-and budget-constrained settings.

Improved reporting of tasks and sub-tasks would enhance understanding of Implementation Mapping processes and how these can be replicated or improved. Clearer guidelines on documenting Implementation Mapping tasks, including prioritisation of determinants and strategy selection, would strengthen the field.

Several elements have been identified in this scoping review which are not formally described in Implementation Mapping yet are commonly reported and seem to improve the implementation process. These include an implementation/planning team, collaborative engagement with stakeholders, the use of questions to guide Implementation Mapping tasks, incorporation of theories, models and frameworks, prioritisation of determinants and implementation strategies and consideration of periodic re-evaluation of the implementation context (40). Incorporation of these elements into future Implementation Mapping projects may also enhance the effectiveness of implementation efforts. Furthermore, extension of the use of questions guiding the Implementation Mapping process beyond the first two tasks to consider subsequent questions such as “*what methods (implementation strategies) could influence the determinants” and “which implementation strategies should be prioritised?”* (Task 3)*; “what implementation materials, resources (etc) will be required”* (Task 4); and *“how can we evaluate process outcomes and implementation outcomes?”* (Task 5) may be beneficial.

## 5 Conclusions and recommendations

Implementation Mapping was developed to help address a highlighted lack of guidance for selection and design of implementation strategies to improve implementation outcomes. It has been applied to a diverse range of interventions in a wide variety of healthcare settings and provides a valuable ‘roadmap’ to guide implementation efforts. The use of theories, models and frameworks featured heavily in most projects, supporting Implementation Mapping as a theory-informed approach. While implementation outcomes were not robustly assessed or reported, Implementation Mapping provides a structured but flexible approach to support the development of implementation strategies and implementation plans which are tailored to context, which may improve the effectiveness of implementation efforts. Approaches for prioritising determinants and strategies in Implementation Mapping are suggested based on the findings and other literature. Finally, key considerations for the use of Implementation Mapping use in future research includes improved methodological reporting, comparative studies of different TMFs and potential core components identified in this review, application across broader geographical settings, and assessment and reporting of both implementation and intervention (clinical) outcomes.
