# Supplementary material for: The use of implementation mapping in healthcare settings: a scoping review
Source: Front Public Health. 2025 Jul 16;13:1603178. doi: 10.3389/fpubh.2025.1603178 (PMC12309394; doi:10.3389/fpubh.2025.1603178)
Supplement: Supplementary file 1 [file Table_1.docx]

Characteristics of included studies

| **Author, year, country, citation** | **Brief objective, concept or Intervention** | **Target population** | **Target health care professionals** | **Implementation stakeholders** | **Clinical or public health intervention** | **Context** | **Setting** | **Healthcare area** |
| --- | --- | --- | --- | --- | --- | --- | --- | --- |
| Bonafide, C. et al. 2022, USA (24) | De-implementation of continuous pulse oximetry monitoring in children hospitalized with bronchiolitis (continuous monitoring is only recommended in specific circumstances). | Pediatric inpatients (2-23 months old) with bronchiolitis in non-intensive care, non-emergency department, non-step down inpatient units. | Hospital clinicians caring for paediatric inpatients with bronchiolitis. | Study team and clinical stakeholders | Clinical | Pediatric Research in Inpatient Settings (PRIS) Network hospitals | Hospital | Paediatrics |
| Creger, T. et al. 2022, USA  (25) | Implementation of evidence-based HIV prevention practices. Cross-agency data sharing to improve the HIV status-neutral care continuum. | People undergoing PrEP screening and PrEP care. | Managers at county health departments, AIDS services organisations, and community-based organisations; clinicians providing PrEP screening and PrEP care at these organisations. | Alabama Department of Public Health; Alabama Quality Management Group (AQMG), a consortium of 13 geographically dispersed Ryan White-funded parts C and D programs. | Public health | County health departments, AIDS services, and community-based organisations | Primary or community care | HIV |
| Dickson, K et al. 2022, USA  (39) | Implementation of two behavioural health EBPs into the existing Care Coordination program serving patients with chronic health conditions (e.g. Diabetes, hypertension): 1. Mental Health First Aid. 2. Adverse Childhood Experiences Screener. | Underserved populations including Hispanic, Latino/a, lower income, racial and ethnic minority groups and uninsured individuals | Care Coordinators, Federally Qualified Health Centre (HQHC) leaders | Key stakeholders at the FQHC, and investigators who have extensive experience working with Hispanic or Latino/a communities. | Public health | Federally Qualified Health Centres | Primary or community care | Mental health |
| Domlyn, A et al. 2022, USA  (40) | Implementation of 5A's, an evidence-based, technology-assisted tobacco cessation tool. | Tobacco users who are patients at a community healthcare centre. | Medical assistants and clinicians. | FQHC clinical and administrative staff: physicians, advanced practice providers, behavioural health specialists, nurses, medical assistants, patient educators, administrative and clinical leaders. Partnered with an academic with expertise in implementation science and clinical psychology. | Public health | Federally Qualified Health Centres | Primary or community care | Substance  Use (tobacco) |
| Fakha, A et al. 2022 & Fakha, A. et al 2023, Netherlands  (30, 31) | *Transitional care innovations to optimise care continuity for older persons when transferring between care settings,* to help meet their care needs, and ultimately improve their quality of life. | Older persons receiving long-term care services in multiple care settings (e.g. Nursing homes, assisted living facilities, homecare). | Leaders of organisations, healthcare professionals (caregivers) | Core research team (with expertise in both transitional care and implementation science) and one additional expert in Implementation Mapping. | Clinical | Not stated | Residential care | Geriatrics |
| Hoskins, K. et al. 2022  (41) | Managed Problem Solving (MAPS+) - an evidence-based intervention for HIV medication adherence and care retention. | People with HIV | Community health workers, clinical team members. | Prescribing clinicians, non-prescribing clinicians, clinic administrators, policymakers, medical case managers, behavioural health consultants. | Clinical | 13 Ryan White-funded HIV clinics serving people with HIV across Philadelphia, Pennsylvania | Clinics | HIV |
| Ibekwe, L. et al. 2022, USA (15) | Delivery of cancer prevention and control (CPC) phone navigation program to increase breast, cervical and colorectal cancer screening; HPV vaccination and smoking cessation among 2-1-1 Texas helpline callers. | Clients calling the 2-1-1 helpline - connects callers with basic health and social services within their communities at no cost. | 2-1-1 Cancer specialists, cancer control navigators, call centre managers | Three 2-1-1 Texas call centres, UT Health researchers. | Public health | 2-1-1 service: confidential telephone service connecting callers to basic health and social services within their local communities at no cost. | Primary or community care | Cancer |
| Juckett et al. 2023, USA (32) | Implementation of interRAI Home Care Frailty Scale assessment by a home-delivered meal agency at baseline (enrolment), 3 months and 6 months. | Home care meal program clients - typically older adults who are unable to safely and independently perform routine mealtime activities | Home-delivered meal staff (a diverse group including social work, nursing, community health, and dietetics), agency leadership. | Home-delivered meal agency, researchers. | Public health | Not-for-profit organisation that providing home-delivered meals and nutritional support services to older adults, age 60 and over, in the five surrounding counties of Columbus, Ohio. | Primary or community care | Geriatrics |
| Jurczuk, M. et al. 2021 UK(51) | OASI care bundle consisting of four primary and secondary prevention practices to reduce severe perineal tearing (OASI) during childbirth. | Pregnant women giving birth in participating hospitals | Not stated | Not stated | Clinical | 20 NHS maternity units | Maternity hospitals | Maternity care |
| Kang, E. et al. 2022 & Kang, E. et al 2023, USA (27, 28) | Implementation of MyGoals: enabling rehabilitation clients to achieve personally meaningful rehabilitation goals by supporting occupational therapists in providing a high-quality and person-centred goal setting and goal management intervention. | Adults with chronic conditions in community-based rehabilitation who did not have severe cognitive or communication impairment. | OTs who were over the age of 18, were licensed OTs and had at least 1 year clinical experience in goal setting and goal management with adults with chronic conditions in community-based rehabilitation settings. Seven OTs participated. | OTs, adults with chronic conditions, research team. | Clinical | Community-based rehabilitation | Primary or community care | Rehabilitation |
| Kennedy, M. et al. 2020, Australia (16) | To facilitate oncologist referrals to a co-located exercise clinic at a cancer treatment centre | Oncology patients | Oncologists, patient services officers, accredited exercise physiologists, billing officers, centre leaders. Operations manager to oversee maintenance | Stakeholder advisory group: 4 exercise oncology researchers, 1 implementation researcher, 3 members of the oncology clinic leadership team, 2 cancer patient representatives | Clinical | Co-located exercise clinic (Co-LEC) at GenesisCare (private oncology clinic) in Western Australia | University-affiliated private oncology clinic | Cancer |
| Klaiman, T. et al. 2020, USA (42) | To incorporate prone positioning as a strategy to reduce mortality in patients with severe ARDS into an overarching response to the COVID-19 crisis | ICU patients with SARS | Bedside clinicians (nurses, respiratory therapists, hospitalists, advanced-practice providers, trainees and critical care specialists) and leaders of participating ICU units | Task force: bedside clinicians and leaders: one ICU director, three critical care nursing leaders, two respiratory therapists, three members of research team | Clinical | 9 ICUs within 4 hospitals of the Penn Medicine Health System and three ICUs of the University of Michigan Medical Centre | Hospital (ICU) - secondary and tertiary | Critical care (ICU) |
| Kurlander et al. 2022, USA (33) | Medication-optimisation strategies to reduce the risk of upper GI bleeding for patients prescribed anticoagulant-antiplatelet therapy: (1) de-prescription of unnecessary antiplatelet therapy (2) use of proton pump inhibitor | Patients co-prescribed anticoagulant and antiplatelet therapy | AMS clinicians (nurses/pharmacists); clinicians within the broader health system (discontinuation/initiation of medications) | Participatory planning group: physicians (gastroenterology, cardiovascular medicine, primary care); patients with anticoagulation experience; clinical pharmacist, anticoagulation service nurses, study coordinator, health IT specialist | Clinical | Michigan Medicine (tertiary academic medical centre). Delivered by the anticoagulation management service (AMS) | Tertiary academic medical centre | Medication safety |
| Li, J. et al. 2021, USA (49) | Implementation of high value care (consistent with clinical practice guidelines) to patients presenting to an ED with syncope. | Patients presenting to an emergency department with syncope | Emergency medicine, hospital medicine, cardiology clinicians and stakeholders (primary care provider, nurse manager, diagnostic test/procedure manager) | Clinicians and managers in relevant departments; primary care providers, nurse managers, diagnostic test/procedure managers | Clinical | A diverse group of health systems and hospitals: an academic medical centre (AMC); an urban faith-based community health system; a not-for-profit health system serving a predominantly rural Appalachian population; and a community teaching hospital in a suburb. | Hospital emergency departments | Critical care (ED) |
| Lovero, K. et al. 2022, Mozambique (50) | Integration of screening (PHQ-A screening tool) and treatment (IPT-AG) for adolescent depression into primary care clinics in Mozambique. | Adolescents with psychiatric disorders | Implementers and adopters: national and local health officials (adolescent and mental health programming), mental health specialists, primary care providers, general medicine technicians, sexual and reproductive health counsellors, nurses, physicians. | Implementation planners: implementation scientists and mental health researchers, policy makers and mental health specialists in Mozambique. | Public health | Community-level primary care clinics in Maputo City, the capital of Mozambique, LMIC. | Primary or community care | Mental health |
| Perez Jolles, M. et al. 2022, USA (46) | Adverse childhood Experiences (ACEs) screening of children in primary care - Californian ACE screening policy ""ACEs Aware" | Children attending primary care clinics | Medical Assistants, Community Health Advisors, Clinic Mangers | Implementation team: Director of Research, Data Manager, Director of Pediatric Programs, Data Coordinator. Researchers, healthcare leadership and implementers, end-users (caregivers of children 0-5). A diverse group of 44 stakeholders: Professional roles included medical doctors, clinic manager, medical assistants, medical scribes, nurses, and technology managers. An additional group of end-users (13 caregivers) also provided feedback on the screening process. | Public health | Five community-based primary care clinics (part of a large FQHC with multiple locations in inland Southern California, serving largely Hispanic/Latinx patients in frontier, rural, semi-urban and urban regions in California | Primary or community care | Mental health |
| Perkison, W. B., et al. 2023, USA (29) | Implementation of the National Diabetes Prevention Program (National DPP) - an evidence-based program designed to reduce diabetes risk through intensive group counselling in nutrition, physical activity, and behavioural management | Patients at participating primary health clinics - primarily serving under or uninsured patients with limited access to healthcare, with prediabetes or risk of developing diabetes | Implementers: staff members responsible for making program referrals and/or clinic administrator responsible for educating staff. Maintainers: clinic leaders from administration, health care providers, National DPP providers | University of Texas Health Science Centre, The Centre for Quality Health IT Improvement at he the School of Biomedical Informatics (UT Health team); Texas Department of State Health Services. Project funded by CDC. Adopters: Clinic leadership and/or staff member/s directly involved in deciding to set up program referral processes | Public health | FQHC with four clinic sites, a rural health centre and two private practices | Primary or community care | Chronic medical conditions |
| Roth I. et al. 2021, USA (17) | Implementation of an evidence-based Integrative Pain Management (IPM) intervention in an outpatient clinic with a high burden of patients with chronic pain. IPM interventions including a range of complementary and integrative health therapies such as mindfulness, yoga, Tai Chi, qigong, and other non-invasive techniques, many of which target chronic pain | Patients with chronic pain | Clinicians, staff, administrators | Implementation team included a physician trained in qualitative methods, a senior implementation scientist, a physician/researcher who developed the IMGV, a physician trained in IPM, three IPM researchers, and a research assistant. Stakeholders: clinic administrators (medical director and financial administrator), physicians, nursing staff, scheduling staff | Clinical | Outpatient setting with a high burden of patients with chronic pain | Outpatient clinics | Chronic pain |
| Savas, L. et al. 2023, USA (34) | To accelerate and improve implementation and maintenance of Salud e Mis Manos (SEMM) - a community health worker-delivered evidence-based intervention to increase breast and cervical cancer screening among medically underserved low-income Latinas | Low-income Latinas | Community health workers | Planning team: researchers and stakeholders (clinic staff, SEMM champions, community health workers). Additional stakeholders: The Breast and Cervical Cancer Collaborative of Texas, representatives of the CDC-funded Texas Prevention Centre Community Advisory Board, incl community leaders representing community-based CHW organisations, community health workers with substantial field experience working with Latinas on breast and cervical cancer screening interventions in FQHCs | Public health | Texas Community Health Centres | Primary or community care | Cancer |
| Schroeck, F. et al 2022 & Zuboff 2024 (under review), USA (478, 48) | To promote risk-aligned bladder cancer surveillance (optimising surveillance for bladder cancer recurrence - to improve over-use among lower risk and under-use among those at higher risk) | Patients known to have a history of bladder cancer (needing ongoing surveillance) | Healthcare provider | Research team. Physician advisory group. Patient advisory group | Clinical | Six Department of Veterans Affairs sites - clinics - within Department of Veteran Affairs. Specifically, bladder cancer clinics | Outpatient clinics | Cancer |
| Thackeray, A et al. 2022, USA (35) | Implementation of Coach2Move, a person-centred physical therapy intervention that has demonstrated success in changing physical activity behaviours among older adults.  Targeting chronic MSK conditions such as low back pain | Target participants: patients who are 50 years and older with a chronic MSK condition | Physical therapists, physical therapy assistants | Implementation planning group: researchers, patient stakeholders, physical therapists, social workers with expertise in motivational interviewing, and Coach2Move developers | Clinical | Academic health system, University of Utah Health (UHealth). Included all 7 outpatient physical therapy clinics within greater Salt Lake City area and Park city. These clinics represent 122 physical therapists and 2 different management structure | Outpatient clinics | Chronic pain |
| Valerio-Shewmaker, M. et al. 2022, USA (52) | Target:BP national initiative of AHA and AMA to improve BP control rates using evidence-based approaches. Unique aspect of Target:BP is focus on building community clinic capacity to implement and maintain guideline-based care and promote accurate hypertension monitoring to improve patient-level outcomes | Patients with hypertension | Healthcare providers, administrative staff, non-physician team members (community health workers, physicians' assistants), nurses | Organisational implementation team: program champion, leadership and others identified at the organisation (generally incl clinic leadership, management and implementers including medical directors, nurse team members, patient navigators, operation managers and IT team members) | Clinical | Four clinics from rural and urban counties in Texas (greater Dallas-Fort Worth and Rio Grande Valley areas) | Primary or community care | Chronic medical conditions |
| Philpot, L. et al. 2023, USA (45) | Digital and mobile (mHealth) eLearning solutions to support individuals with centrally sensitised chronic pain | Patients across Mayo Clinic system with an active digital connection to electronic health record (HER) and diagnosed with an applicable chronic pain condition by any practicing clinician at Mayo Clinic | Caregivers: Physicians, nurses, pharmacists, therapists | Sponsors: funders, institutional leaders. Program leaders: conceptualisers, day-to-day leaders. Program enablers: technical and clinical experts. Adopters: physicians, nurses, pharmacists, therapists | Clinical | Mayo Clinic - Integrated healthcare delivery organisation | Integrated healthcare delivery organisation - hospitals and clinics | Chronic pain |
| Allen et al. 2023, USA  (44) | Establishment of a Hereditary cancer Clinic, providing management strategies for individuals identified at high risk for hereditary cancers | Individuals identified to be at high risk for hereditary cancers | Caregivers within the clinics (Care specialists) | Implementation team members (4), specialists (9), patients with cancer genetic pathological variants (11) | Public Health | Hereditary Cancer Clinic at the Medical University of Southy Carolina | Outpatient clinic | Cancer |
| Farris, P. et al. 2023, USA (43) | Implementation of a tool to Improve research capacity for cancer prevention and control studies in rural areas. Community-engaged approach to co-creation of the tool | Patients in rural areas | Healthcare professionals preparing cancer research applications; rural community healthcare professionals and stakeholders reviewing research proposals | Research team (implementation planners). Rural community stakeholders, divided into two workgroups (each consisting of 4-6 community participants) | Public Health | Rural primary and community care (cancer prevention and control) | Primary and community care | Cancer |
| Collins et al. 2022, USA (26) | Program to improve medication adherence for oral anticancer agents (for leukaemia treatment) | People with leukaemia | Pharmacists, nursing team, physician/nurse physician/physician assistant, MAP technician, social worker | Advisory panel: 9 clinicians, 7 administrators, 2 patients | Clinical | Academic and community cancer care | Two settings: an academic cancer centre and a community cancer centre | Medication safety |
| Fritz et al. 2024, USA (36) | Telehealth delivery of nonpharmacologic care from physical therapists to persons with chronic back pain receiving care in community health centres (CHCs). Two EBIs: a brief consult and an extended PT program, delivered across two treatment phases | Persons with chronic back pain receiving care in Community Health Centres | Cancer researchers, Community research advisory committee, primary care practitioners, oncologists | CHC leaders/providers from 7 organisations | Clinical | Nine CHC organisations in Utah serving urban and rural communities providing primary care to all persons regardless of ability to pay. | Primary or community care | Chronic pain |
| Hohmeier et al. 2024, USA (37) | Optimised care model: community pharmacy practice using task delegation to expand clinical service delivery | Patients having prescribed medications dispensed at a pharmacy | Pharmacy staff including pharmacy technicians, pharmacists | Research team; working group included small group of pharmacists, university researchers; pharmacy champion, pharmacy leadership | Clinical | University of Tennessee Health Science Centre and a single division of a nationwide supermarket pharmacy chain with 6 community pharmacies | Community pharmacies | Pharmacy |
| Juckett et al. 2024, USA (38) | I-STROM (Implementation STRategies for Outcome Measurement) - a bundle of implementation strategies to support use of standardised outcome measures to objectively evaluate upper extremity function in a rehabilitation setting for people who experience a stroke | People who have experienced a stroke | Stroke rehabilitation practitioners (occupational therapists) | Partnered with a large academic health system serving>200 stroke patients annually through intensive rehabilitation care | Clinical | Academic health system/intensive rehabilitation care | Rehabilitation | Rehabilitation |
